# Comparison of SARS-CoV-2 Antibody Response by Age Among Recipients of the BNT162b2 vs the mRNA-1273 Vaccine

**DOI:** 10.1001/jamanetworkopen.2021.24331

**Published:** 2021-09-02

**Authors:** Nathan E. Richards, Behnam Keshavarz, Lisa J. Workman, Michael R. Nelson, Thomas A. E. Platts-Mills, Jeffrey M. Wilson

**Affiliations:** 1 Division of Allergy and Clinical Immunology, University of Virginia, Charlottesville

## Abstract

This cohort study compares antibody responses in a cohort in which both BNT162b2 and mRNA-1273 COVID-19 vaccines were administered.

## Introduction

Two COVID-19 mRNA vaccines, BNT162b2 (ie, Pfizer/BioNTech) and mRNA-1273 (ie, Moderna), were approved via the US Food and Drug Administration Emergency Use Authorization (FDA-EUA) for adults in December 2020. Both incorporate mRNA that encodes for the prefusion stabilized spike glycoprotein, use a prime-boost strategy, and have shown strong immunogenicity in preclinical and clinical studies.^1,2^ Although the 2 vaccines share similar features and both showed strong efficacy in clinical trials, there are formulation differences, and there has been little head-to-head evaluation of antibody responses. In this cohort study, we used a quantitative assay for IgG to SARS-CoV-2 spike-receptor binding protein to compare antibody responses in an employee cohort in which both BNT162b2 and mRNA-1273 were administered. We hypothesized that there could be differences in antibody levels elicited by the 2 vaccines and explored the effect of age on immunogenicity.

## Methods

This cohort study was approved by the University of Virginia institutional review board. Written informed consent was provided. This study followed the Strengthening the Reporting of Observational Studies in Epidemiology (STROBE) reporting guideline.

Adults affiliated with the University of Virginia, the majority of whom were employed by the Health System, were recruited to participate in this study. The analysis included all individuals who received 2 doses of either BNT162b2 or mRNA-1273 and had a blood sample drawn 7 to 31 days after the second vaccine (ie, postboost). Of note, the vaccine that was received depended on local availability. Some recipients also had a baseline (ie, within 7 days prior to the first vaccine) or preboost (ie, 14-28 days after the first vaccine but preceding the second vaccine) blood sample collected. Serum was isolated and IgG to SARS-CoV-2 spike RBD and nucleocapsid were measured with a quantitative ImmunoCAP-based system using a Phadia 250 (Thermo-Fisher/Phadia), as previously described.^3^ Statistical analysis was performed with GraphPad Prism 8 (GraphPad Software). Antibody levels were expressed by geometric mean with 95% CIs and comparisons were made between groups with the Mann-Whitney U test. To account for multiple comparisons, *P* < .0125 was considered significant. Additional methods are available in the Supplement. Statistical analysis was done in June 2021.

## Results

The median age of the 167 recipients was 42 (interquartile range, 32-57 years), with 63 recipients (38%) aged 50 years or greater; 120 recipients (72%) were women (Table). There were no differences in age, sex, or race between those who received BNT162b2 (n = 79) or mRNA-1273 (n = 88). Only 6 (4%) of the participants had serologic evidence of prior COVID-19. Levels of IgG to SARS-CoV-2 spike RBD were lower in recipients of BNT162b2 as compared with mRNA-1273 at both the preboost blood draw (5.9 μg/mL [95% CI, 3.7-9.6 μg/mL] vs 19.1 μg/mL [95% CI, 15.8-23.1 μg/mL]) and postboost blood draw (45.9 μg/mL [95% CI, 37.0-57.0 μg/mL] vs 68.5 μg/mL [95% CI 61.9-75.7 μg/mL]) (Figure). Recipients aged 50 years and older who received BNT162b2 had preboost IgG levels (2.1 μg/mL [95% CI, 1.0-4.3 μg/mL]) that were lower than levels in recipients younger than 50 years who received BNT162b2 (10.2 μg/mL [95% CI, 6.0-17.5 μg/mL]) and also as compared with age-similar peers who received mRNA-1273 (14.7 μg/mL [95% CI, 10.0-21.2 μg/mL]) (Figure). Recipients aged 50 years and older who received BNT162b2 had postboost IgG levels (31.1 μg/mL [95% CI, 19.9-48.7 μg/mL]) that were lower than the levels in younger recipients of BNT162b2 (59.0 μg/mL [95% CI, 48.8-71.4 μg/mL]) and age-similar peers who received mRNA-1273 (71.8 μg/mL [95% CI, 58.1-88.8 μg/mL]) (Figure).

**Table.** Characteristics of Vaccinated Study Population

Characteristics	No. (%)			*P* value^a^
	Total (n = 167)	Pfizer/BNT162b2 (n = 79)	Moderna/mRNA-1273 (n = 88)	
Age, median (IQR), y	42 (32-57)	43 (34-57)	40 (31-57)	.21^b^
Age, y				
<50	104 (62)	48 (61)	56 (64)	.70
≥50	63 (38)	31 (39)	32 (36)	.70
Sex				
Female	120 (72)	57 (72)	63 (72)	.94
Male	47 (28)	22 (28)	25 (28)	.94
Race/ethnicity^c^				
White	130 (78)	61 (77)	69 (78)	.85
Black	15 (9)	8 (10)	7 (8)	.62
Asian	16 (10)	6 (8)	10 (12)	.41
Other^d^	6 (3)	4 (5)	2 (2)	.33
Baseline samples	40 (24)	22 (28)	18 (20)	.26
Preboost samples	70 (42)	38 (48)	32 (36)	.12
Postboost samples	167 (100)	79 (100)	88 (100)	>.99
Serologic evidence of prior COVID-19 infection^e^	6 (4)	5 (6)	1 (1)	.07

^a^ χ^2^ test was used for all statistical significance tests unless otherwise noted.

^b^ The *t* test was used for statistical significance.

^c^ Race/ethnicity was self-reported and collected to evaluate as a variable in vaccination responses.

^d^ Other includes Hispanic, Native American, Pacific Islander, and all other races. Hispanic was listed as an option on the questionnaire. However, the number of individuals who identified as Hispanic was small, and we included those individuals in the other category.

^e^ Defined by IgG to spike receptor binding protein >2.5μg/mL at baseline or IgG to nucleocapsid >5μg/mL in any sample.

**Figure.**
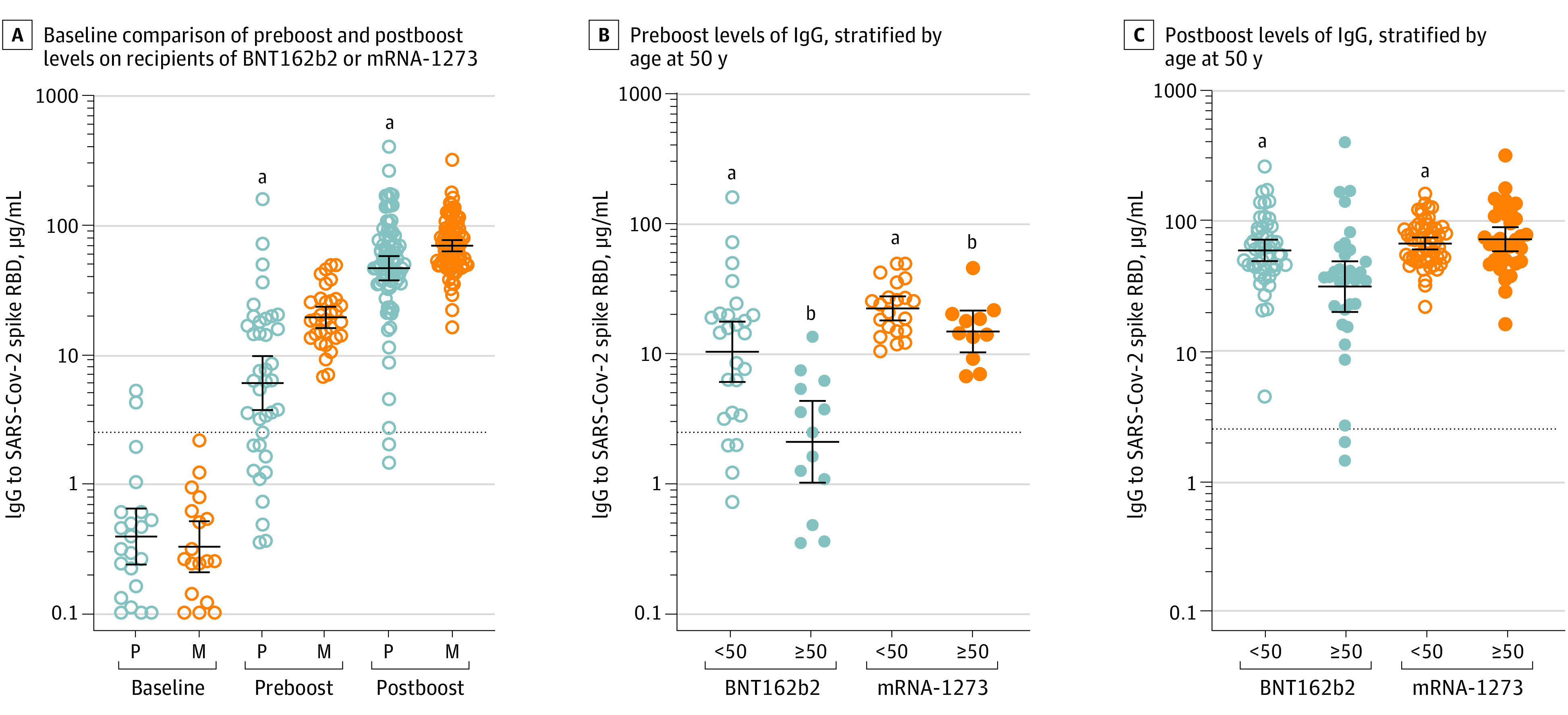
Levels of IgG to SARS-CoV-2 Spike RBD in an Employee Vaccine Cohort Preboost is 14 to 28 days after the primary immunization and postboost is 7 to 31 days after the boost immunization. Data are presented as geometric means with 95% CI and comparisons with Mann-Whitney U test. The dotted lines denotes cutoff of assay, as previously described.^3^ Accounting for multiple comparisons, *P* < .0125 was considered as significant. ^a^*P* < .001. ^b^*P* < .05.

## Discussion

In this cohort study, we used a quantitative assay and found that BNT162b2 elicited relatively lower antibody levels in older adults vs younger adults, which is consistent with emerging reports.^4,5^ By contrast, there was no difference in postboost antibody levels in older adults vs younger adults who received mRNA-1273. One explanation for the difference in immunogenicity observed in older adults could relate to the amount of mRNA used in the respective vaccines, with 30 μg contained in BNT162b2 and 100 μg in mRNA-1273.^1,2^ A limitation of this study is that the neutralizing antibodies were not measured; however, several groups have reported a strong correlation between SARS-CoV-2 binding and neutralizing antibodies.^6^ Additional studies are warranted to determine whether binding antibodies to SARS-CoV-2 can be used to predict clinical protection against COVID-19.

## Notes

Supplement.
eMethods.
