# Supplementary material for: Comparison of SARS-CoV-2 Antibody Response by Age Among Recipients of the BNT162b2 vs the mRNA-1273 Vaccine
Source: JAMA Netw Open. 2021 Sep 2;4(9):e2124331. doi: 10.1001/jamanetworkopen.2021.24331 (PMC8414189; doi:10.1001/jamanetworkopen.2021.24331)
Supplement: Supplement. — eMethods. [file jamanetwopen-e2124331-s001.pdf]

Supplemental Online Content

Richards NE, Keshavarz B, Workman LJ, Nelson MR, Platts-Mills TAE, Wilson JM. Comparison of SARS-CoV-2 antibody response by age among recipients of the BNT162b2 vs the mRNA-1273 vaccine. *JAMA Netw Open*. 2021;4(9):e2124331.
doi:10.1001/jamanetworkopen.2021.24331

eMethods.

This supplemental material has been provided by the authors to give readers additional information about their work.

© 2021 Richards NE et al. *JAMA Network Open.*

Supplemental Methods

Details of study

The study was commenced on December 18, 2020, targeting adult subjects affiliated with the University of Virginia (UVA) who were planning to or who had recently received a COVID-19 vaccine. The majority of participants worked for the UVA Health System and were eligible to receive vaccines during the months between December 2020 and March 2021 as part of the hospital vaccination campaign. Both BNTB162b2 and mRNA-1273 were administered during this campaign. The selection of the mRNA vaccine that was administered to any given individual reflected local availability at the time of their first injection. Vaccine eligibility was initially restricted to frontline healthcare workers, but by late December vaccine eligibility was open to most members of the healthcare system. To our knowledge there are no subjects in the current cohort who chose which mRNA vaccine they received. Recruitment into the study was conducted by flyers posted across the medical campus and by listing on the UVA clinical trials website. All subjects signed a written consent, filled out a questionnaire on history of COVID-19 exposure and co-morbid health conditions, and provided a 6 mL sample of blood by venipuncture. Serum was isolated by standard procedure and stored at -30C. Antibodies were assayed in batched samples approximately every two weeks. The primary objective was to measure antibodies between 7 and 31 days following the second vaccine (post-boost blood draw), as this timeframe was expected to reflect peak antibody levels. In a subset of participants, a blood sample was obtained during a baseline interval (within 7 days prior to the first vaccine) or pre-boost interval (between 14-28 days after the first vaccine, but preceding the second vaccine). Subjects who were recruited but did not have blood drawn within the pre-defined post-boost interval were excluded from this analysis. In most cases the lack of

© 2021 Richards NE et al. *JAMA Network Open.*

samples as the baseline and pre-boost interval reflected the fact that many subjects became aware of the study after they had already received at least one vaccine.

Details of antibody assays

All samples were assayed for IgG to SARS-CoV-2 spike RBD and for IgG to SARS-CoV-2 nucleocapsid using the ImmunoCAP-based biotin-streptavidin technique, as previously described[1]. Each sample was also assayed with a control unconjugated streptavidin ImmunoCAP which served as an internal control to subtract out background binding. The assay has a quantitative read-out in µg/mL and the internal calibrator has been previously validated using an anti-SARS-CoV-2 spike RBD monoclonal IgG (clone CR3022)[2]. Evidence of prior COVID-19 infection was defined by IgG to spike RBD > 2.5µg/mL at baseline or IgG to nucleocapsid > 5µg/mL in any sample. The cut-off for the spike RBD assay was determined in a prior report investigating antibody levels in pre-COVID-19 serum samples and samples from patients admitted to hospital with COVID-19[1]. The cut-off for the nucleocapsid assay here was increased from the 2.5µg/mL cut-off we reported previously. This was done to account for a modest increase in IgG to nucleocapsid that we observed in many samples that had paired data and also batch-to-batch variability in the antigen preparation.

Supplemental References

1. Keshavarz B, Wiencek JR, Workman LJ, Straesser MD, Muehling LM, Canderan G, et al. Quantitative Measurement of IgG to Severe Acute Respiratory Syndrome Coronavirus-2 Proteins Using ImmunoCAP. Int Arch Allergy Immunol 2021:1-8.
2. Tian X, Li C, Huang A, Xia S, Lu S, Shi Z, et al. Potent binding of 2019 novel coronavirus spike protein by a SARS coronavirus-specific human monoclonal antibody. Emerging Microbes & Infections 2020; 9:382-5.

© 2021 Richards NE et al. *JAMA Network Open.*
